# Counseling patients and family members in out-of-hospital emergency situations: a survey for emergency staff

**DOI:** 10.1186/s12912-017-0205-7

**Published:** 2017-02-23

**Authors:** Eija Paavilainen, Riitta Mikkola, Mari Salminen-Tuomaala, Päivi Leikkola

**Affiliations:** 1 School of Health Sciences, University of Tampere/ Hospital District of South Ostrobothnia, 33014 Tampere, Finland; 2 School of Health Sciences, University of Tampere, Tampere, Finland; 3 School of Health Care and Social Work, Seinäjoki University of Applied Sciences, Seinäjoki, Finland; 4 Hospital District of South Ostrobothnia, Seinäjoki, Finland

**Keywords:** Out-of-hospital emergency care, Counseling, Patients, Family members

## Abstract

**Background:**

Not much is known about emergency care delivered in patients’ homes or other out-of-hospital settings. This study aims to describe out-of-hospital emergency staff’s experiences of encountering and counseling patients and their family members.

**Methods:**

A descriptive cross-sectional design was applied. Data were collected from a hospital district in Finland from emergency care staff via an electronic survey questionnaire specifically developed for this purpose (*N* = 125, *N* = 142 reponse rate 59%, response rate 53%) and analyzed using descriptive statistics.

**Results:**

Respondents succeeded in encountering (up to 3.88/4) and counseling (up to 3.89/4) patients and family members. Challenges were related to introducing themselves to family members (3.20/4), to interacting with patients from different cultures (3.38/4) and to allowing family members to be present in care situations (2.29/4). Providing emotional support (2.56/4), especially to family members, and confirming (3.16/4) and ensuring continuity of care instructions (3.00/4) were found to be challenging.

**Conclusions:**

High-level counseling in acute out-of-hospital situations demands that care providers can put themselves into the patient’s and family’s situation, ensure follow-up care and provide care instructions to both patients and families. The presence and participation of family members is essential in acute care situations outside hospital contexts. Ensuring that these contents are included and practiced during basic and continuing emergency care education for nurses and other emergency staff is crucial for developing counseling practices.

## Background

In-hospital emergency care has been, in contrast to out-of-hospital emergency care, extensively studied in the past few years. Research has focused on counseling [1, 2], information giving, the presence of family members [3], staff’s coping with fears [4] and staff’s clinical skills [5]. It has been shown [2] that both patients and their families appreciate the presence of family members at the emergency unit as an important part of care; more than 90% of patients and family members have been found to agree with the importance of having family members present at the unit. According to a follow-up study conducted in an emergency department [2], it is crucial to systematically develop possibilities for the presence of family members as an integral part of good care and counseling.

The presence of the patient’s family is even more pronounced in acute situations which occur outside the hospital, before or without transportation to the hospital emergency unit. Family members of the acutely ill person are very often present and the ones who call the ambulance. In such situations, success in encountering and counseling patients and families is a starting point for the care process and crucially important to ensure good quality care.

This study focuses on the emergency care process in the patient’s home or other non-clinical setting, from the viewpoint of encountering the patient and family members present in the situation. There is no earlier research evidence on the topic. The following paragraphs summarize research evidence about encountering and counseling patient and families in similar short acute care situations in clinical settings.

High quality counseling given in emergency circumstances has been characterized as one whose contents meet the patient’s needs and expectations [6], the family members’ needs for information and support [7] and also the emergency staff’s notions of what patients should know to cope with self-care at home [8]. It has been proposed that counseling should start with the patient’s life situation and context [9]. A literature review on patient and family counseling [10] suggests that high-level counseling has the following elements: patient-centeredness and individualization; a wide variety of counseling methods; provision by various professionals; good interaction and high quality provision of information.

Family members have been reported to expect information about the patient’s situation to be able to keep up to date and assist the patient, both in the acute situation at the emergency unit, but also afterwards during self-care at home [6]. Investigators have revealed, for example, that family members expect information about the patient’s illness, examinations, pharmacological care, and also about the patient’s current status and prognosis [11–13]. Some studies [14] have disclosed family members’ wish to receive more information about their possibilities to participate in the patient’s care, both in the acute stage and later at home. As suggested in several studies, development of interaction between nursing and other staff and family members is necessary to make family members’ involvement possible and also to make it easier for the staff to provide cognitive and emotional support [15–17].

The presence of family members can make it possible to develop counseling practices towards more patient and family-centered discussions and to deepen the structure and content of counseling. This would mean a shift from traditional mechanical and uniform information-giving to patients only towards information and support provided to patients and family members together, based on their individual needs. This type of counseling, carried in the form of a discussion, considers the patients’ life situation as a whole. Counseling based on the patient’s life situation means involving both the patient and family members in the care and counseling process, according to the family’s current information and care needs. (cf. [18]). This is possible also in short-term, acute care situations occurring in emergency care outside the hospital setting, provided that the action is well-planned [10, 18].

As previously stated, not very much is known about out-of-hospital emergency care, provided in the patient’s home or any other site of an incident or emergency. In Finland, emergency medical services are strictly governed by legislation and practically organized by hospital districts according to pre-defined service levels. Law also governs the qualification and competence levels of emergency care providers, who have been trained to assess the patients’ needs for care and either treat them on site or transport them to hospital by an ambulance. If necessary, emergency care providers can help arrange alternative transport to a healthcare unit.

Staff who work in out-of-hospital emergency care have to independently care for acutely ill patients in need of urgent care [19]. Advising and counseling patients and supporting them throughout the acute situation are essential elements of the work [20]. A study proposes that to be able to assess patient status and needs for care, care providers working in out-of-hospital settings require situational sensitivity, as well as ability to make independent decisions and implement care in rapidly changing circumstances. Nursing staff also need to be able to cope with challenging interaction situations and work in unfamiliar environments [20].

It follows from the above that there is a genuine need to develop out-of-hospital emergency care by encouraging family presence and more patient and family-centered counseling as an important part of the care. More effective care, delivered in the patient’s home or other setting outside hospital, will improve quality and possibly cut down on care expenditure [21, 22].

This study is part of a larger research project dealing with statistical modeling, evaluation and follow-up of out-of-hospital emergency care, including staff’s clinical skills as well as encountering and counseling of patients and families. The project incorporates the perspectives of staff, patients and family members. The knowledge derived can be used to systematically develop the quality of out-of-hospital emergency care of a very broad mixture of acute situations following emergency calls, and to make the care more family-centered.

This paper presents baseline data, aiming to describe out-of-hospital emergency staff’s experiences of encountering and counseling patients and their family members in acute situations, and seeking to answer the following research question: How do out-of-hospital emergency staff encounter and counsel patients and their family members in acute care situations? While this paper presents staff’s experiences, the ultimate aim is to use the knowledge to better meet patients’ needs and expectations and family members’ needs for information and support. This forms the basis of high-level encountering and counseling.

## Methods

Data were collected by electronic questionnaires sent to all emergency staff members (*N* = 238; 125 registered nurses and 113 others: emergency medical technician/hospital & ambulance attendant or practical nurse) of a hospital district in Finland in 2014. The hospital district represents a rather typical setting in the Finnish healthcare system. It provides advanced medical care services, including emergency care, for a geographical region. The hospital district under study serves a population of approximately 200, 000. The electronic questionnaire was pre-tested in a pilot study (*N* = 17) in June and July. As no amendments were required, the results of the pilot study were included within the major results, obtained in autumn 2014. The number of responses was 125 combined with the 17 pilot study responses, or a total of 142. The response rate was 59%. The research project obtained all the relevant research permissions and ethical approval from Pirkanmaa Hospital District Ethics Committee (no. R13164H). The participants were recruited voluntarily, based on their informed consent.

### Instrument

The questionnaire was specifically developed for this study. To achieve the best possible inter-rater reliability, a group of experts including emergency care leaders, emergency staff, an emergency care teacher and researchers contributed to the questionnaire design. The instrument was based on carefully conducted searches of earlier research and literature [4, 23–32] on elements discovered in high-level encountering and counseling of patients and families. The instrument was pilot tested to find out how respondents understood the questions and to obtain feedback for any ambiguous points on the questionnaire. No feedback was received, so there was no need to change anything. During data collection, respondents first answered background questions (items 1–8) to provide information about their age, sex, qualification, current position and type of employment, as well as their work experience in the current position and in emergency care and health service in general. The background questions were followed by statements that concerned encountering (10 statements) and counseling (12 statements) patients and their family members. A 7-point Likert scale with the following options was used: 1 = not part of my role definition; 2 = totally disagree; 3 = disagree; 4 = somewhat disagree; 5 = somewhat agree; 6 = agree and, 7 = totally agree. In addition, staff’s experiences of counseling were explored by an open question in which respondents were asked to write what else they would like to say about encountering and counseling patients and family members.

### Analysis

SPSS (Statistical Package for the Social Sciences) for windows 22.0 was used for statistical analysis. In regard to age, respondents were classified into the categories <25, 25–34, 35–44 and >45 years. They were further classified as follows: basic or advanced level emergency care position; registered nurse or other qualification, and permanent or fixed term work contract. Last, the following categories were used for work experience: Experience in the current position <1.5, 1.5–2.4 and 2.5/>2.5 years, experience in emergency care and experience in the health service <3, 3–8 and >8 years.

Responses to the items on encountering and counseling patients and family members were classified into five categories as follows: 0 = not part of my role definition; 1 = disagree or totally disagree; 2 = somewhat disagree; 3 = somewhat agree; and 4 = agree or totally agree. In the final printout, 0 or not part of my role definition was classified as missing information. However, encountering and counseling were naturally included in all respondents’ work; the whole emergency staff encounter and counsel patients and their family members. This meant that respondents responded to questions concerning the topic as a matter of course. The results were analyzed using frequency distributions, means, standard deviation, cross tabulation and principal axis factoring. When classifying the background variables it was ensured that there was an adequate number of observations in each category and that the categories remained comparable. The overall response rate was 59% and the respondents were well representative of all age groups and care provider categories. Very few items remained unanswered (see Tables 1, 2, 3, 4 and 5), showing the importance of encountering and counseling as a part of emergency care.

**Table 1:** Respondent Demographics

Respondents’ background (*N* = 142)	*N* (%)
Age (*n* = 142)	
Under 25 years old 25–34 years old 35–44 years old Over 45 years old	23 (16.2) 55 (38.7) 40 (28.2) 24 (16.9)
Sex (*n* = 142)	
Female Male	71 (50) 71 (50)
Current job (*n* = 142)	
Basic level emergency care Advanced level emergency care	93 (65.5) 49 (34.5)
Qualification (*n* = 142)	
Emergency Medical Technician/Hospital & Ambulance	56 (39.4)
Attendant or Practical Nurse	
Nurse	86 (60.6)
Employment (*n* = 142)	
Permanent On contract	106 (74.6) 36 (25.4)
Work experience in current position (*n* = 141)	
Less than 1.5 years 1.5 years–2.4 years 2.5 or more	33 (23.4) 76 (53.9) 32 (22.7)
Work experience in health services (*n* = 140)	
Less than 3 years 3–8 years 8 years or more	25 (17.8) 55 (39.3) 60 (42.9)

**Table 2:** Patient and Family Encounters

Encountering patients and family members	Mean	Median	SD	Lowest value^a^	Highest value^a^
My work is based on ethical values (*N* = 141)	3.88	4.00	0.348	2	4
I plan each patient contact individually (*N* = 142)	3.70	4.00	0.593	1	4
I introduce myself to the patient (*N* = 142)	3.20	3.00	0.853	1	4
I introduce myself to the family member (*N* = 142)	2.99	3.00	0.922	1	4
I explain the patient the reasons for the procedures I carry out (*N* = 142)	3.87	4.00	0.342	3	4
I am able to put myself in the patient’s life situation (*N* = 142)	3.63	4.00	0.689	1	4
I attend to the various needs of patients from different cultures (*N* = 142)	3.38	3.50	0.722	1	4
The presence of the patient’s family member is inconvenient for me in the emergency care situation (*N* = 142)	2.29	2.00	1.001	1	4
I make an effort to provide the patient enough information about his/her current condition (*N* = 142)	3.84	4.00	0.423	1	4
I make an effort to provide the family member enough information about the patient’s current condition (*N* = 142)	3.81	4.00	0.411	2	4

^a^1 disagree/totally disagree; 2 somewhat disagree; 3 somewhat agree; 4 agree/totally agree

**Table 3:** Contents of sum variables for encountering patients and family members

Variable	Communality	Loading	% of variance explained	Cronbach’s alpha
1. Introducing oneself			18.0	.90
Introducing oneself to the patient	.749	.913		
Introducing oneself to the family member	.882	.813		
2. Individual patient contact			16.6	.63
Ethical value foundation	.215	.410		
Individual planning of the patient contact	.323	.544		
Putting oneself in the patient’s life situation	.350	.556		
Attending to various needs of patients from different cultures	.393	.594		
Explaining nursing procedures to the patient	.391	449		
3. Providing information			14.8	.60
Providing information about the patient’s condition to the patient	.473	.679		
Providing information about the patient’s condition to the family member	.684	.811

**Table 4:** Counseling patients and family members

Counseling patients and family members	Mean	Median	SD	Lowest value^a^	Highest value^a^
I have good communication skills (*N* = 142)	3.87	4.00	.362	2	4
I give the patient’s home care instructions orally (*N* = 142)	3.92	4.00	.378	1	4
I give the patient’s home care instructions in writing (*N* = 141)	1.43	1.00	.796	1	4
I have enough time to go over home care instructions (*N* = 141)	3.30	3.00	.808	1	4
I make sure that the patient has understood the home care instructions (*N* = 141)	3.77	4.00	.526	1	4
I make sure that the family member has understood the home care instructions (*N* = 141)	3.72	4.00	.539	2	4
I support the patient psychologically (*N* = 141)	2.74	3.00	.907	1	4
I support the family member psychologically (*N* = 142)	2.54	2.50	.926	1	4
I provide health education (*N* = 142)	3.03	3.00	.858	1	4
I encourage the family member to participate in the patient’s follow-up care (*N* = 141)	3.16	3.00	.782	1	4
I inform patients where to contact in case of further problems (*N* = 141)	3.89	4.00	.318	3	4
I find arranging follow-up care challenging (*N* = 139)	3.00	3.00	.985	1	4

^a^1 disagree/totally disagree; 2 somewhat disagree; 3 somewhat agree; 4 agree/totally agree

**Table 5:** Contents of sum variables for counseling patients and family members

Variable	Communality	Loading	% of variance explained	Cronbach’s alpha
1. Psychological support			15.8	.89
Psychological support to the patient	.883	.931		
Psychological support to the family member	.835	.886		
2. Understanding instructions			15.2	.83
The patient understands instructions	.901	.913		
The family member understands instructions	.742	.785		
Going over instructions	.292	.362		
3. Health promotion and supporting follow-up care			13.0	.76
The family member’s participation in follow-up care	.998	.983		
Health education	.444	.584		
4. Counseling patients			11.7	.64
The patients’ problem situations	.466	.572		
Instructions in writing	.349	.587		
Instructions orally	.585	.709		

The chi-square test (×^2^) was used to examine statistical associations among variables in cross tabulation. The purpose of principal axis factoring was to examine if the total variation of the variables actually reflected variation in a lower number of factors. Cronbach’s alpha was also calculated to assess the reliability of the sum variables (cf. [33]). Values > .60 were considered to indicate reliability of the instrument. Responses to the open question on the development of counseling for no-transport situations were analyzed using inductive content analysis. First, the investigator read the data carefully to become familiar with it. The analysis continued by reducing, clustering and abstracting the data, according to the research question. The inter-rater reliability was assured within the research team. One of the team members conducted the basic analysis, after which the remaining members examined the original data to verify the analysis. The research team discussed the results and unanimously agreed that they were correct. The results of the qualitative analysis were used to support the quantitative results.

The demographic data on the respondents are presented in Table 1. The contents for encountering and counseling patients and family members are reported using means in Tables 2 and 4 and percentages in the text. The contents of the sum variables for encountering and counseling patients and family members are presented in Tables 3 and 5, with the respective percentages provided in the text. The comparison between demographic categories (age, work experience etc.) showed no statistically significant differences and the data are therefore not presented. The results for qualitative data are reported in connection with quantitative results on counseling.

## Results

Respondents were 19–58 years old (mean 34) and there was an equal number of men and women. The majority (61%) of the informants were registered nurses and the rest were emergency medical technicians, hospital and ambulance attendants and practical nurses. Most respondents (75%) held a permanent position, while the rest had a fixed-term contract. The majority (77%) of all respondents had less than 2 years’ experience of their current work (mean 1.9 years). The mean for work experience in emergency health service in general was 8.3 years (Table 1).

### Encountering patients and family members

Almost all (98.5%) respondents agreed with the statement that their work was founded on ethical values. Similarly, the majority (94%) reported that they planned each patient contact individually. It was further revealed that 19% of the informants did not introduce themselves to patients, and 25% did not introduce themselves to family members. All respondents replied that they explained to their patients the reasons for the emergency procedures. The majority of them agreed fully or to some extent with the statement that they were able to put themselves into the patient’s position, whereas 8% disagreed. Approximately half of the informants marked the option “totally agree” to report that they attended to the needs of patients from different cultures; the other half disagreed to varying extent. As regards the presence of family members in care situations, 45% of the informants found it inconvenient. Over 80% of all respondents agreed with the statement that they made an effort to provide both patients and family members adequate information concerning the patient’s status.

### Counseling patients and family members

Most respondents (87%) agreed that they had good communication skills. Almost all of them (95%) gave home care instructions orally and less than 10% gave the instructions in writing. Less than half (45%) of the informants felt that they had adequate time to go over the home care instructions with patients or family members. Over 80% of all respondents ascertained that the patient and family member had understood the instructions. Approximately one fifth (21%) reported that they had adequate time to support the patient psychologically in the emergency care situation, whereas the rest of the informants disagreed. Half of all respondents announced that they did not have time to support the family member psychologically. The majority, however, encouraged family members to participate in the patient’s follow-up care and all respondents made sure that patients knew where to contact in case of further problems. Almost one third (28%) thought that arranging follow-up care was challenging.

The qualitative analysis revealed that respondents found counseling patients a challenging but essential part of the emergency care situation. Counseling was considered especially demanding, if staff members could not be certain that the instructions had been understood correctly, if the recipients were unwilling to have instructions, or if the patient and family member had different opinions about the no-transport decision. Respondents wished for more counseling material in writing and guidelines on how to respond to a repeat contact from the same address. Written material was required especially for the care of burns, wounds, fever, flu and gastroenteritis. It was also suggested that families should be given a general leaflet on where to contact if the patient should deteriorate. Respondents further proposed that the entire population should be better informed of the nature and purpose of emergency medical services, especially as regards situations in which an ambulance is or is not required. It was suggested that co-operation between emergency units and doctors on call should be intensified and telephone counseling improved by providing another contact where patients and families could call when in uncertainty or need for care instructions. According to the respondents, emergency care staff might benefit from clearer guidelines for no-transport situations. Sometimes there is no need to transport the patient to the hospital, but the acute situation can managed on site. The patient, often assisted by a family member, can manage at home with help of home care instructions. Further training on counseling was also suggested to improve individual counseling of patients and care providers’ personal counseling skills in general.

Below is an example of what care providers said about developing their counseling skills:

“For my own part, I could give more thought to how I present things to them, and make sure that the patient understands the situation. I should certainly pay more attention to finding the right words, instead of just handing them a piece of paper and wishing them goodnight.”

## Discussion

This study provides valuable insight into emergency staff experiences from encountering and counseling patients and their family members within their homes, outside the usual acute care context. It is often decided that the patient should not be not transported to hospital, which poses another special challenge to follow-up care and counseling. Still, several elements are the same as in any counseling situation: the importance of successful oral and written communication, provision of information, and attending to the circumstances at hand. Attention given to the individual’s needs, family, life situation and culture has been found to contribute to good counseling, as revealed by a literature review [10]. This study indicates that emergency staff need to further develop their skills in encountering patients and families, and become better aware of patients’ and family members’ different needs in counseling, for example of those resulting from the patient’s cultural background. Care providers can also be advised to always introduce themselves to patients and family members. It is possible that the combination of an acute situation and presence of several people in an out-of-hospital context brings new elements to encountering and counseling patients and families, if we compare the situation to the work carried out in hospital circumstances, on terms of the hospital.

Many care providers who took part in this study found the presence of family members inconvenient in the emergency situation. Other studies (e.g. [10, 13]) have confirmed that the family members’ presence can be challenging, especially if the staff are not aware of the advantages of family presence or if they do not provide adequate support or information to the family members. However, the care providers in this study reported having provided adequate information to both patients and family members, and also having ensured that the information was understood. The finding may be related to the out-of-hospital context, where family members possibly tend to be more active than in hospital circumstances. Secondly, it has been found [34–36] that the family members’ fear, anxiety and restlessness in the home context may render the situation more inconvenient and challenging for care providers. Respondents in this study also found arranging follow-up care to be demanding, although they wished to encourage the family’s involvement in the care.

Finally, this study revealed that providing psychological support was seen as a demanding activity in emergency situations. This may be understandable, considering that the care providers’ main focus is on managing the acute situation. Still, there is no denying that family members, often inevitably present in the care situation, also require at least a modicum of attention, if not earlier, then at least after the acute situation has been resolved.

### Methodological limitations

As no suitable instrument was available for this study, a questionnaire was developed based on knowledge of acute care competencies and of counseling patients and family members in general. The research team consisted of experienced researchers and the questionnaire was pre-tested. The instrument is based on international research finding, according to which the elements of counseling can be considered rather universal [10]. The actual data collection process covered all emergency staff members in a major hospital district. The response rate was 59% and the respondents were well representative of the population. Results can be generalized to the entire geographical area covered in this study. Even beyond that, they can be useful in national and international efforts to develop out-of-hospital emergency care.

### Implications

Besides a wide range of acute care competencies, out-of-hospital emergency care requires preparedness to encounter and counsel patients and their families. The elements of high-level counseling are mainly universal, but their successful application to acute out-of-hospital situations demands putting oneself in the patient’s and family member’s situation and paying special attention to the provision of care instructions to both patients and family members to ensure continuous care. The presence and participation of family members is essential. Good organization and implementation of emergency care services rests on a solid national and regional foundation and effective basic and continuous education programs. Last, patients’ and family members’ experiences must be heard and included in training programs, to be practiced during basic and continuing emergency staff education. This is crucial for developing counseling practices.

## Conclusions

This study shows that care providers’ competencies in encountering and counseling patients and family members need to be developed to reach higher-level practice in out-of-hospital emergency care. Family members are very often involved in out-of-hospital emergency care situations; they are the ones who call for help and are present when the ambulance arrives and when care is delivered. They will stay with the patient after emergency care providers have left. The counseling provided to them must help ensure patient safety and continuity of care.

## Abbreviations

SPSS: Statistical Package for the Social Sciences
VTR: Valtion Tutkimusrahoitus (in English: State Research Funding)

## References

[1] Paavilainen E, Salminen-Tuomaala M (2010). Web-based learning environment as a means for continuing nursing education in hospital: the course implemented for emergency unit staff. J Nurses Staff Dev.

[2] Paavilainen E, Salminen-Tuomaala M, Leikkola P. Counseling for patients and family members: a follow-up study in the emergency department. ISRN Nurs. 2012; Article ID 303790, doi:10.5402/2012/303790. 10.5402/2012/303790 PMC3447345 23008782

[3] Nikki L, Lepistö S, Paavilainen E (2012). Experiences of family members of elderly patients in the emergency department: a qualitative study. Int Emerg Nurs.

[4] Mikkola, R. Henkilökunnan kokema pelko ja selviytymiskeinot ensiapupoliklinikoilla: malli pelosta selviytymisestä. [Experiences of fear and coping strategies among staff in emergency departments: model for coping with fear.] Academic dissertation. University of Tampere; 2013. http://urn.fi/URN:ISBN:978-951-44-9137-5. Accessed 12 July 2016.

[5] Salminen-Tuomaala M, Leikkola P, Mikkola R, Paavilainen E (2015). Workers’ clinical skills at out-of-hospital emergency care. Emerg Med.

[6] Maxwell KE, Stuenkel D, Saylor C (2007). Needs of family members of critically ill patients: a comparison of nurse and family perceptions. Heart Lung.

[7] Rantanen A, Heikkilä A, Asikainen P, Paavilainen E, Åstedt-Kurki P (2010). Perheiden tukeminen elämänkulun eri vaiheissa. [Support received by families in health care.] Hoitotiede. Finnish NursSci J.

[8] Sun BC, Adams JG, Burstin HR (2001). Validating a model of patient satisfaction with emergency care. Ann Emerg Med.

[9] Donovan HS, Ward S (2001). A representational approach to patient education. J Nurs Scholarsh.

[10] Raitanen K, Kylmä J, Paavilainen E (2015). Short-term patient and family counseling for acute health change—an integrative literature review. Clin Nurs Stud.

[11] Bond AE, Draeger CR, Mandleco B, Donnelly M (2003). Needs of family members of patients with severe traumatic brain injury. Implications for evidence-based practice. Crit Care Nurse.

[12] Eggenberger SK, Nelms TP (2007). Being family: the family experience when an adult member is hospitalized with a critical illness. J Clin Nurs.

[13] Paavilainen E, Salminen-Tuomaala M, Kurikka S, Paussu P (2009). Experiences of counseling in the emergency department during the waiting period: importance of family participation. J Clin Nurs.

[14] Lee LY, Lau YL (2003). Immediate needs of adult family members of adult intensive care patients in Hong Kong. J Clin Nurs.

[15] Attree M (2001). Patients’ and relatives’ experiences and perspectives of ‘good’ and ‘not so good’ quality care. J Adv Nurs.

[16] Auerbach SM, Kiesler DJ, Wartella J, Rausch S, Ward KR, Ivatury R (2005). Optimism, satisfaction with needs met, interpersonal perceptions of the healthcare team, and emotional distress in patients’ family members during critical care hospitalization. Am J Crit Care.

[17] Verhaeghe S, Defloor T, Van Zuuren F, Duijnstee M, Grypdonck M (2005). The needs and experiences of family members of adult patients in an intensive care unit: a review of the literature. J Clin Nurs.

[18] Wright LM, Leahey M (1999). Maximizing time, minimizing suffering: the 15-minute (or less) family interview. J Fam Nurs.

[19] Cooper J, Grant J (2009). New and emerging roles in out of hospital emergency care: a review of the international literature. Int Emerg Nurs.

[20] Salminen-Tuomaala M, Leikkola P, Paavilainen E (2014). Patient and staff safety incidents and near misses in out-of-hospital emergency care. Emerg Med.

[21] Brice JH, Garrison HG, Evans AT (2000). Study design and outcomes in out-of-hospital emergency medicine research: a 10-year analysis. Prehosp Emerg Care.

[22] El Sayed MJ (2012). Measuring quality in emergency medical services: a review of clinical performance indicators. Emerg Med Int.

[23] Badir A, Sepit D (2007). Family presence during CPR: a study of the experiences and opinions of Turkish critical care nurses. Int J Nurs Stud.

[24] Bernard SA, Nguyen V, Cameron P, Masci K, Fitzgerald M, Cooper, DJ, . . . Judson R. Prehospital rapid sequence intubation improves functional outcome for patients with severe traumatic brain injury: a randomized controlled trial*.* Ann Surg. 2010; 252, 959–65*.* 10.1097/SLA.0b013e3181efc15f 21107105

[25] Campeau A. Why paramedics require”Theories-of-Practice”. Australasian Journal of Paramedicine 2012; 6 (2). http://scholar.google.fi/scholar_url?url=http://ajp.paramedics.org/index.php/ajp/article/download/451/451&hl=fi&sa=X&scisig=AAGBfm1GXI3BMIyzJqFSQkxSUy4zft-GJQ&nossl=1&oi=scholarr&ved=0ahUKEwin5LjwtZnSAhUB3SwKHaYqA_AQgAMIHSgAMAA. Accessed 26 Jan 2015.

[26] Deakin CD, King P, Thompson F (2009). Prehospital advanced airway management by ambulance technicians and paramedics: is clinical practice sufficient to maintain skills?. Emerg Med J.

[27] Evans R, Mcgovern R, Birch J, Newbury-Birch D (2013). Which extended paramedic skills are making an impact in emergency care and can be related to the UK paramedic system? a systematic review of the literature. Emerg Med J.

[28] Hjälte L, Björn-Ove S, Herlitz J, Karlberg I (2007). Why are people without medical needs transported by ambulance? a study of indications for pre-hospital care. Eur J Emerg Med.

[29] Mason S, Knowles E, Freeman J, Snooks H (2008). Safety of paramedics with extended skills. Acad Emerg Med.

[30] Nirel N, Goldwag R, Feigenberg Z, Abadi D, Halpern P (2008). Stress, work overload, burnout, and satisfaction among paramedics in Israel. Prehosp Disaster Med.

[31] O’Meara PF, Tourle V, Stringling C, Walker J, Pedler D (2012). Extending the paramedic in the rural Australia: a story of flexibility and innovation. Rural Remote Health.

[32] Woollard M. The role of the paramedic practitioner in the UK. Australasian Journal of Paramedicine 2006; 4(1). https://ajp.paramedics.org/index.php/ajp/article/view/357. Accessed 26 Jan 2015.

[33] Burns N, Grove SK (2005). The practice of nursing research: conduct, critique & utilization.

[34] Salminen-Tuomaala M, Leikkola P, Mikkola R, Paavilainen E (2015). Factors that influence the counseling of family members in out-of-hospital emergency medical care. Fam Med Sci Res.

[35] Zavala S, Shaffer C (2011). Do patients understand discharge instructions?. J Emer Nurs.

[36] Palonen M, Kaunonen M, Åstedt-Kurki P (2016). Family involvement in emergency department discharge education for older people. J Clin Nurs.

